# The Deep Learning Evolution in Wireless Physical Layer Communications: Applications, Challenges, and Evolutionary Directions

**DOI:** 10.3390/s26113609

**Published:** 2026-06-05

**Authors:** Hang Xu, Yin Liang, Rui Xie, Yang Kong

**Affiliations:** 1 School of Information Engineering, Nanchang University, Nanchang 330036, China; 5801123035@email.ncu.edu.cn (H.X.); 5702124009@email.ncu.edu.cn (Y.K.); 2 School of Resources and Environment, Nanchang University, Nanchang 330036, China; 5801123034@email.ncu.edu.cn

**Keywords:** artificial intelligence, deep learning, wireless communication physical layer, channel estimation, 6G

## Abstract

With the continuous evolution toward sixth-generation (6G) wireless communication systems, emerging scenarios such as terahertz transmission, integrated sensing and communication (ISAC), and ultra-massive multiple-input multiple-output (MIMO) have significantly increased the complexity, nonlinearity, and uncertainty of wireless propagation environments. The conventional model-driven paradigm, established upon Shannon information theory and precise mathematical modeling, is increasingly constrained by model-mismatch issues in real-world deployments. This paper systematically reviews recent advances in deep learning-enabled physical-layer signal processing. We examine intelligent channel estimation, signal detection, and end-to-end communication systems based on autoencoder architectures. We then analyze key technical challenges—including interpretability, data dependence, computational complexity, privacy and security in distributed learning, and system-level performance-overhead trade-offs—along with state-of-the-art solution strategies such as deep unfolding, transfer learning, model compression, federated learning, and lightweight design. Future evolutionary directions toward AI-native 6G networks, integrated sensing-communication-computing architectures, and intelligent reconfigurable wireless environments are discussed. Furthermore, emerging generative AI techniques, including diffusion models, are identified as a promising direction for addressing data scarcity and enhancing system adaptability. The study demonstrates that hybrid intelligence—integrating model-based prior knowledge with data-driven learning—will become the dominant design philosophy for next-generation intelligent physical-layer systems.

## 1. Introduction

### 1.1. Research Background

Physical-layer signal processing in wireless communications is undergoing a profound paradigm transformation driven by artificial intelligence (AI) [1,2]. From first-generation (1G) to fifth-generation (5G) mobile communication systems, physical-layer technologies have long followed a model-driven paradigm grounded in Shannon information theory. This paradigm relies on constructing precise mathematical models of wireless channels, noise characteristics, and modulation processes, and subsequently designing signal processing algorithms with explicit analytical formulations. Due to the evolutionary nature of mobile communication systems, each generation has been built upon its predecessors, continuously introducing key technologies such as advanced multiple-access schemes to steadily enhance overall system performance [3].

Looking toward sixth-generation (6G) mobile communications, the deployment of emerging scenarios—including terahertz-band transmission, integrated sensing and communication (ISAC), and ultra-massive antenna arrays—has introduced unprecedented complexity, time variation, and uncertainty into signal propagation environments [4,5]. Constructing accurate and engineering-friendly universal channel models under such conditions has become extremely challenging. Consequently, traditional algorithms designed under idealized assumptions often suffer from severe “model mismatch” problems in practical deployments, resulting in significant performance degradation and becoming a critical bottleneck restricting further system performance improvement [2].

Studies have shown that massive multiple-input multiple-output (mMIMO), a core technology for 5G and beyond (B5G), faces inherent challenges such as pilot contamination, high channel estimation complexity, and hardware impairments, which further exacerbate the limitations of model-driven approaches [6]. By deploying hundreds of antennas at base stations, mMIMO systems achieve high spectral efficiency. However, traditional signal processing methods relying on precise channel modeling struggle to effectively address complex scenarios involving multi-user interference and time-varying channels. This limitation has become one of the key driving forces behind the transition of physical-layer research from a model-driven paradigm toward a data-driven paradigm. The integration of machine learning and deep learning techniques provides new optimization pathways for core tasks such as channel estimation, signal detection, and precoding in mMIMO systems, serving as a crucial enabling technology for addressing the above challenges.

### 1.2. Motivation

Against this backdrop, data-driven AI approaches provide a new avenue for addressing long-standing technical challenges. Research has demonstrated that AI models represented by deep learning possess strong capabilities for learning high-dimensional nonlinear features, enabling them to implicitly extract statistical patterns of channel impairments and intrinsic signal structures from large volumes of measured data rather than relying solely on potentially mismatched predefined models [1,7].

This core concept—enhancing or partially replacing traditional modeling through data and learning—is driving a fundamental paradigm shift in physical-layer research from purely model-driven approaches toward data-driven and model–data hybrid methodologies [8].

It is worth noting that current frontier research has moved beyond using AI to optimize individual functional modules and is gradually advancing toward constructing more generalized intelligent physical-layer architectures. For example, recent surveys indicate that emerging multiple-access technologies such as rate-splitting multiple access (RSMA) are considered potential key technologies for 6G, while AI techniques are being employed to optimize complex processes such as precoding [9]. This demonstrates that AI is increasingly regarded as a key enabling technology for realizing the vision of fully intelligent 6G networks integrating communication, sensing, and computing. Its powerful generalization and integration capabilities provide new insights into building intrinsically intelligent future communication networks.

Recently, generative artificial intelligence (GAI) has emerged as a transformative paradigm for physical-layer design. A comprehensive survey by Huynh et al. [10] systematically reviewed GAI applications in physical layer communications, covering channel estimation, equalization, intelligent reflecting surfaces, and joint source-channel coding, highlighting its potential to address longstanding challenges through data generation and distribution learning.

### 1.3. Contributions

This paper aims to systematically review research progress in AI-enabled physical-layer signal processing for wireless communications. The main contributions are summarized as follows: (1) The driving forces behind the paradigm transition from model-driven to data-driven physical-layer design are analyzed, clarifying the inevitability of this transformation in addressing key technological bottlenecks of 6G systems; (2) The application mechanisms and performance advantages of deep learning in core tasks—including intelligent channel estimation, signal detection, and end-to-end communication systems—are comprehensively reviewed; (3) Key challenges currently faced in this field—including interpretability, data dependence, computational complexity, privacy and security in distributed learning, and system-level performance-overhead trade-offs—are thoroughly examined, and state-of-the-art solutions are discussed; (4) Future research directions toward 6G, including AI-native network design, semantic communications, and intelligent integrated sensing–communication–computing frameworks, as well as emerging generative AI and diffusion model-based techniques, are explored.

### 1.4. A Three-Stage Evolutionary Perspective

Surveying the trajectory of AI integration into wireless physical-layer communications, one can discern a clear evolutionary arc: the fusion of AI and physical-layer technologies is progressing from localized enhancement toward systemic reconstruction, and ultimately toward native integration. To provide a coherent organizational logic for this review, we structure the surveyed literature into three progressive stages:

Stage I: Module-Level Replacement. In this stage, deep neural networks are deployed to replace or augment individual functional modules—most notably channel estimation and signal detection—within the conventional transceiver architecture, while preserving the overarching modular framework. The primary objective is performance enhancement within existing system boundaries. This stage represents the most mature body of research, with several techniques approaching practical deployment. Section 2.1 and Section 2.2 survey representative works in this category.

Stage II: System-Level Reconstruction. Moving beyond modular optimization, end-to-end autoencoder architectures treat the entire transmitter–receiver chain as a single differentiable neural network amenable to joint training. This approach challenges the foundational separation principle of conventional communication theory—namely, that source coding, channel coding, and modulation can be designed independently without loss of optimality. While conceptually more radical, this stage grapples with heightened challenges in interpretability, generalization, and hardware compatibility. Section 2.3 surveys developments in this category.

Stage III: Network-Level Native Intelligence. In the 6G vision, AI transcends its role as an external optimization tool to become a constitutive element of network architecture itself. AI-native network designs, semantic communications, and the convergence of sensing, communication, and computing exemplify this shift from “applying AI to communications” toward “building communications upon AI.” Section 4 explores these emerging paradigms.

The five core challenges examined in Section 3—interpretability, data dependence, computational complexity, privacy and security, and performance–overhead trade-offs—cut across all three stages, though their manifestations and severity vary with the depth of AI integration. This three-stage framework not only organizes the exposition but also reveals the key technical transitions that define the field’s evolution.

While several surveys have addressed aspects of AI in physical-layer communications [1,2,10], the present work differentiates itself in the following respects. First, it adopts a unified three-stage organizational lens—module-level replacement, system-level reconstruction, and network-level native intelligence—that organizes previously separate research efforts along a clear technical progression. While the progression from module-level optimization to system-level reconstruction to native AI integration has been discussed in fragmented forms in prior surveys, the present work is the first to employ this three-stage lens as a consistent analytical device that systematically structures the entire review—from the organization of technical sections, through the identification of cross-cutting challenges, to the articulation of 6G paradigms—thereby transforming a descriptive taxonomy into an integrated analytical narrative. Second, it provides cross-task comparative analyses that explicitly contrast methods for channel estimation, signal detection, and end-to-end systems in terms of their performance, computational complexity, and practical deployability. Third, it offers a more critical perspective on emerging topics such as diffusion models and large AI models, candidly discussing their practical limitations alongside their potential, rather than presenting them primarily as promising solutions. Fourth, the survey covers the latest developments through 2026, including generative AI and AI-native 6G paradigms, with a significant proportion of references from 2024 to 2026. These elements collectively provide a more structured and differentiated synthesis than existing surveys on this topic.

### 1.5. Organization

The remainder of this paper is organized as follows. Section 2 reviews the applications of deep learning in key physical-layer tasks, including intelligent channel estimation, signal detection, and end-to-end communication systems. Section 3 analyzes the core challenges faced by AI-enabled physical-layer design and discusses feasible technical solutions. Section 4 explores emerging AI-enabled scenarios and new paradigms for 6G systems. Finally, Section 5 concludes the paper and outlines future research directions.

## 2. Applications of Deep Learning in Key Physical-Layer Tasks

This section surveys AI applications in core physical-layer tasks, covering Stage I module-level replacement (channel estimation and signal detection) and Stage II system-level reconstruction (end-to-end communication systems). The conventional modular architecture of wireless physical-layer systems is increasingly being reshaped by the end-to-end representation learning capability of deep neural networks. The pioneering work of O’Shea and Hoydis [1] demonstrated that communication systems can be modeled as differentiable end-to-end learning frameworks, thereby enabling joint optimization across traditionally separated modules.

### 2.1. Intelligent Channel Estimation

Channel estimation is a fundamental component of coherent wireless receivers. The accuracy of channel state information (CSI) directly determines system reliability and spectral efficiency. Traditional channel estimation methods, such as least squares (LS) and minimum mean square error (MMSE), are derived under linear Gaussian assumptions. Although optimal under ideal conditions, their performance deteriorates significantly when practical channel conditions deviate from modeling assumptions [11,12].

Deep learning offers a data-driven alternative by formulating channel estimation as a nonlinear regression problem. Gao et al. [13] demonstrated that convolutional neural networks (CNNs) can effectively exploit the two-dimensional time–frequency structure of pilot signals. By learning spatial correlations in the received signal grid, CNN-based estimators achieve robust denoising and reconstruction performance even under low signal-to-noise ratio (SNR) conditions and limited pilot overhead. In essence, this approach learns a nonlinear approximation of the MMSE estimator without explicitly requiring precise channel statistics.

**Figure 1:**
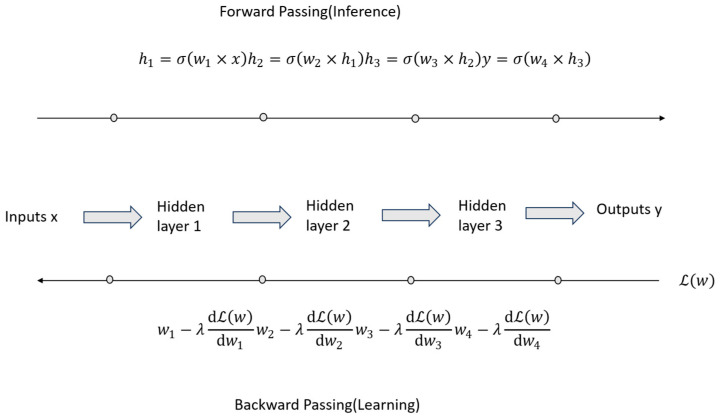
Illustration of the learning and inference processes of a 4-layer CNN. Adapted from [14].

To better understand why CNNs can effectively extract the time-frequency features of wireless channels, Figure 1 illustrates the learning and inference process of a typical 4-layer CNN. As shown in the figure, during forward propagation, the input data (e.g., a two-dimensional pilot signal grid) is sequentially processed by multiple convolutional layers and nonlinear activation functions. Each convolutional layer extracts local features using learnable filters (kernels) and generates feature maps; the activation function (e.g., ReLU) introduces nonlinearity, enhancing the model’s representational capability. The final output layer produces prediction results according to the task objective (e.g., channel estimation). During backpropagation, the gradients of the loss function with respect to the weights of each layer are computed, and network parameters are updated using gradient descent, enabling the model to automatically learn the optimal feature extraction method from training data. This mechanism allows CNNs to implicitly learn the spatial correlations of the channel and the noise distribution, thereby approximating the optimal estimator without requiring precise statistical priors.

For highly dynamic scenarios, recurrent neural networks (RNNs) and long short-term memory (LSTM) architectures have been introduced to capture temporal channel variations. Liao et al. [15] showed that RNN-based channel estimators leverage historical CSI sequences to predict future channel states, achieving superior performance in fast time-varying environments compared with traditional Kalman filtering techniques.

In millimeter-wave massive MIMO systems, channel sparsity and high-dimensionality introduce additional challenges. Zheng et al. [16] proposed a residual learning and multi-path feature fusion network (RL-MFF-Net) for sparse channel reconstruction. By treating quantized received pilot signals as low-resolution images, the framework employs image super-resolution concepts to recover high-precision CSI. Residual connections mitigate gradient vanishing issues in deep networks, while multi-path feature fusion enhances robustness by integrating multi-scale features. Simulation results demonstrate that RL-MFF-Net achieves approximately 4 dB performance gain in low-SNR regions compared with ChannelNet and DnCNN. Moreover, accurate channel reconstruction is maintained even with only eight pilot symbols, significantly reducing pilot overhead.

Robust channel estimation in non-ideal environments has also been extensively investigated. Soltani et al. [17] demonstrated that deep learning-based estimators exhibit strong robustness not only under Gaussian noise but also in the presence of non-Gaussian impulsive interference. In parallel, hybrid model–data-driven approaches have emerged as promising solutions. By embedding domain knowledge into neural architectures, these methods improve both interpretability and data efficiency.

He et al. [18] proposed OAMP-Net2, which unfolds the orthogonal approximate message passing (OAMP) iterative MIMO detection algorithm into a trainable deep neural network. Although primarily designed for detection, its architecture exemplifies the deep unfolding paradigm, where traditional iterative algorithms are mapped into layered neural structures with a limited number of learnable parameters. This hybrid design preserves physical interpretability while enhancing robustness and performance.

**Figure 2:**
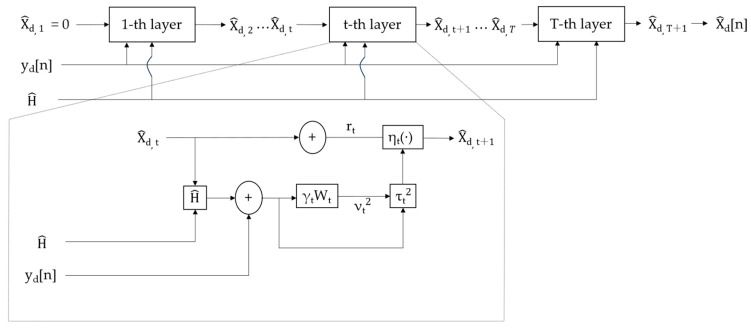
Architecture of the OAMP-Net2 detector. Adapted from [18].

Figure 2 demonstrates how a traditional iterative algorithm (OAMP) can be unfolded into a deep network consisting of T layers with identical structures. Each layer includes a linear estimator, a nonlinear estimator, and an error estimator, along with trainable parameters. This architecture deeply integrates model-driven algorithmic priors with data-driven parameter optimization and represents a typical paradigm for constructing interpretable and efficient hybrid intelligent signal processors.

Very recently, diffusion models—a class of generative AI—have been applied to high-dimensional channel estimation. Zhou et al. [19] proposed a generative diffusion model that significantly outperforms conventional deep learning methods in massive MIMO scenarios, achieving a 10× reduction in estimation delay and halving the pilot overhead.

Similarly, Fu and Si [20] developed a conditional denoising diffusion-based channel estimator specifically for fast time-varying MIMO-OFDM systems. Their method demonstrated superior tracking capability compared to recurrent neural network-based approaches under challenging conditions such as limited pilots and cyclic prefix absence.

Beyond point-to-point estimation, diffusion models have also been applied to massive access scenarios. Recent work [21] demonstrated a generative diffusion model for joint active user detection, channel estimation, and data detection in massive MIMO systems.

The preceding survey reveals distinct performance–complexity trade-offs across different architectural choices for channel estimation. CNN-based estimators excel at capturing local time–frequency correlations with modest computational requirements, but their capacity to model long-range channel dependencies remains constrained by the limited receptive field inherent to convolutional operations. RNN and LSTM architectures effectively track temporal channel evolution and are particularly suited for high-mobility scenarios; however, gradient vanishing and limited parallelizability during training remain persistent challenges. Deep unfolding methods achieve a favorable balance between interpretability and data-driven adaptation by embedding domain knowledge into trainable network layers, yet their structural design is tightly coupled to the specific iterative algorithm being unfolded. Diffusion models demonstrate remarkable reconstruction fidelity under extremely low SNR conditions, but their iterative inference procedure incurs substantial latency, typically requiring tens to hundreds of denoising steps per estimation. This fundamentally conflicts with the sub-millisecond latency budgets of URLLC applications. While acceleration techniques such as denoising diffusion implicit models (DDIM) and knowledge distillation can reduce the number of sampling steps, the computational overhead remains orders of magnitude higher than that of feed-forward neural networks. Consequently, diffusion-based channel estimation is most appropriately positioned for offline or delay-tolerant scenarios rather than as a blanket replacement for conventional estimators.

From a system-level perspective, two key trade-offs warrant continued investigation. The first concerns the pilot overhead versus estimation accuracy trade-off: while deep learning methods consistently outperform conventional estimators under limited pilot resources, the optimal pilot density under non-stationary channel conditions remains an open problem. The second pertains to accuracy versus computational complexity: high-fidelity approaches demand computational resources that may exceed the capabilities of edge devices. Promising directions for future research include lightweight diffusion architectures with reduced inference steps and adaptive pilot pattern design informed by channel statistics.

Additionally, as 6G moves toward extra-large MIMO (XL-MIMO) arrays with significantly expanded apertures, the near-field regime becomes increasingly relevant for channel estimation. Unlike conventional far-field channels, where planar wavefronts can be assumed, near-field channels exhibit spatial non-stationarity—different regions of the array observe substantially different propagation characteristics—and coupled angular-distance features that standard far-field methods handle poorly. Addressing these unique challenges represents an important direction for future research. Recent work has explored hierarchical pre-selection and multi-level dynamic threshold strategies to cope with these non-stationary sparsity patterns, offering a practical pathway for near-field channel estimation in XL-MIMO systems [22].

To facilitate a systematic comparison, Table 1 summarizes the representative channel estimation methods discussed in this section.

### 2.2. Intelligent Signal Detection

Signal detection in multi-antenna systems is computationally demanding. The optimal maximum likelihood (ML) detector achieves minimum error probability but incurs exponential complexity with respect to antenna number and modulation order. Linear detectors such as zero-forcing (ZF) and linear minimum mean square error (LMMSE) offer reduced complexity but suffer significant performance loss in high-interference scenarios [18,23].

**Figure 3:**
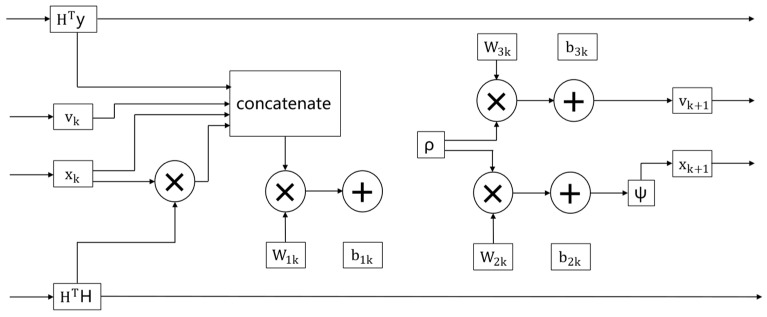
Architecture of a single layer in the DetNet deep MIMO detector. Adapted from [23].

Deep learning reformulates signal detection as either a classification or regression problem, thereby bypassing computationally intensive matrix inversion operations. Samuel et al. [23] introduced a deep neural network-based detector, termed DetNet, that directly learns the nonlinear mapping from received signal vectors to transmitted symbols. The detector is derived by unfolding a projected gradient descent algorithm for the ML optimization problem, where each iteration of the algorithm is mapped to a layer in the neural network. Figure 3 illustrates the internal structure of a single DetNet layer. As shown, each layer takes as input the matched-filter output $H^{T}y$, the current estimate ${\hat{x}}_{k}$, the feedback term $H^{T}H{\hat{x}}_{k}$, and an auxiliary variable $v_{k}$. Through a combination of linear transformations, ReLU activations, and a learnable soft sign function, the layer produces the updated estimate ${\hat{x}}_{k+1}$ and updated auxiliary variable $v_{k+1}$. This architecture enables the network to learn optimal step sizes and shrinkage parameters during end-to-end training, resulting in near-ML performance under nonlinear distortion and interference conditions with significantly reduced computational complexity.

For massive MIMO detection, deep unfolding techniques have demonstrated remarkable effectiveness. By converting iterative algorithms such as approximate message passing (AMP) into multi-layer neural networks and training a small set of parameters end-to-end, detection complexity is reduced from exponential to polynomial order while maintaining near-optimal bit error rate (BER) performance [24]. This makes real-time implementation feasible.

In addition to structural optimization, label encoding strategies significantly influence detection performance. Techniques such as one-hot encoding per antenna (OHA) and direct symbol encoding (DSE) transform high-dimensional detection problems into structured multi-class classification tasks, achieving favorable trade-offs between accuracy and computational efficiency [25]. These strategies enable lightweight detection models suitable for edge devices.

Deep learning-based MIMO detectors occupy distinct positions along the performance–complexity spectrum. DetNet achieves near-ML detection performance by learning optimal step sizes from data, effectively compressing what would require tens of classical iterations into approximately ten learned layers; however, its performance degrades noticeably when the channel matrix becomes ill-conditioned. Deep unfolding methods derived from approximate message passing provide theoretical convergence guarantees under i.i.d. channel assumptions, yet their efficacy in spatially correlated channels remains less thoroughly validated. Classification-based approaches substantially reduce computational requirements, but their scalability to massive MIMO configurations is constrained by the exponential growth of output classes with modulation order and antenna count.

A noteworthy direction for future investigation is the development of adaptive detection architectures capable of dynamically selecting among detectors of varying complexity based on instantaneous channel quality and user load. Furthermore, the majority of existing studies assume perfect channel state information at the receiver, neglecting the inevitable propagation of channel estimation errors into the detection stage. Joint channel estimation and detection frameworks may offer a path toward mitigating this performance bottleneck.

Table 2 provides a comparative overview of the key signal detection methods.

### 2.3. End-to-End Communication Systems

Moving beyond the replacement of individual functional modules, end-to-end communication systems based on autoencoder architectures represent a more radical paradigm shift [26]. In this framework, traditional transmitter components (coding and modulation) and receiver components (demodulation and decoding) are replaced by a unified deep neural network trained jointly in an end-to-end manner. Early studies even attempted to use a single deep neural network to perform all processing tasks of an OFDM receiver, implicitly enabling joint optimization across modules [12].

Recently, Elfiky et al. [27] provided a tutorial review of end-to-end deep learning in wireless systems, covering autoencoder-based transceiver design, scalability challenges, and adaptation to dynamic channel conditions.

**Figure 4:**
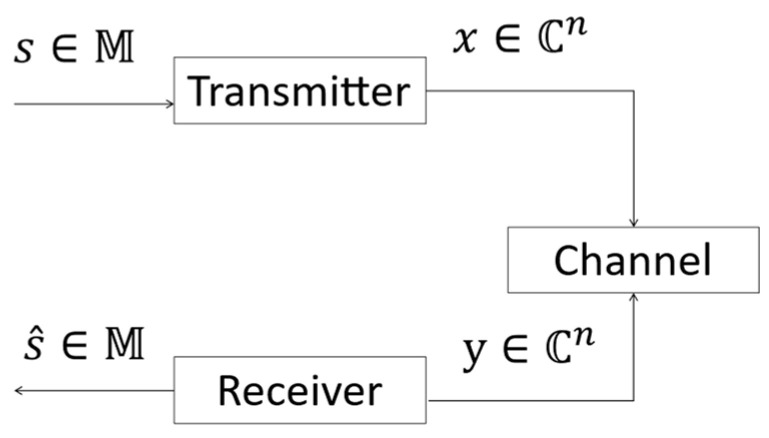
Communication system schematic. Adapted from [27].

**Figure 5:**
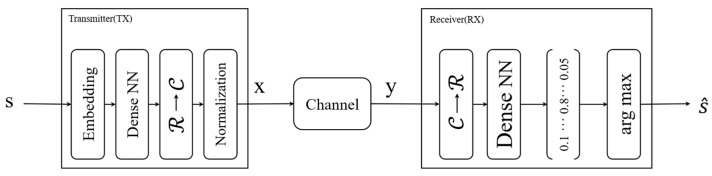
Autoencoder architecture. Adapted from [26].

Figure 4 illustrates a simplified communication system consisting of a transmitter, channel, and receiver [27]. In deep learning-based end-to-end systems, this process is further modeled as an autoencoder structure (Figure 5), where the transmitter and receiver correspond to the encoder and decoder, respectively, and the channel is modeled as a differentiable noise layer. This architecture enables end-to-end optimization of the entire system through gradient-based learning with the objective of minimizing the overall bit error rate.

A complementary survey by Islam and Shin [28] focused specifically on data-driven end-to-end PHY systems from the perspective of enabling semantic applications across multiple modalities. While their work provides a detailed review of the E2E-semantic interface, the present survey adopts a broader scope, covering channel estimation, signal detection, and 6G paradigms under a unified three-stage evolutionary framework.

Through end-to-end training, the transmitter and receiver are jointly optimized to minimize system-level error rates. The transmitter network functions as a nonlinear encoder that learns to map input bit streams directly into complex baseband signal waveforms. This process is equivalent to learning an “intelligent modulation” scheme that is deeply adapted to specific channel conditions. The receiver network acts as a decoder responsible for recovering original information directly from signals corrupted by channel impairments [29].

Such global optimization has the potential to discover transmission schemes that go beyond the performance limits of traditional modular architectures derived from Shannon theory. However, purely black-box designs suffer from inherent limitations in interpretability. To address this issue, deep unfolding networks provide an effective compromise by transforming iterative steps of traditional communication algorithms into neural network layers. This embeds known communication model priors into learnable architectures, improving performance while preserving physical interpretability [24].

Christopoulou et al. [30] proposed an autoencoder-based end-to-end framework that integrates channel coding, demodulation, and modulation recognition modules into a unified “coding–estimation–classification” optimization pipeline. Experimental results demonstrate that such deeply collaborative design significantly reduces bit error rates caused by interference and distortion within specific frequency bands, validating the potential of end-to-end learning to overcome performance bottlenecks inherent in traditional modular communication system design.

End-to-end autoencoder architectures represent a conceptually elegant departure from modular design, offering the theoretical possibility of discovering transmission schemes that surpass conventional performance limits. Nevertheless, several formidable barriers impede their translation from simulation to practical deployment. First, the interpretability gap is particularly acute: learned signal constellations and waveforms often defy human understanding, complicating standardization efforts and system debugging. Second, generalization fragility poses a severe challenge—autoencoders trained on specific channel statistics frequently exhibit catastrophic performance degradation when confronted with even modest deviations from the training distribution. Third, hardware compatibility concerns arise from the fact that end-to-end optimization may produce waveforms that conflict with the linearity requirements of practical RF chains.

Deep unfolding networks offer a pragmatic compromise by imposing modular structural priors while retaining end-to-end trainability, thereby achieving partial interpretability without sacrificing performance. Looking forward, the integration of autoencoder principles with model-based modular receivers—alongside the development of robust training methodologies that explicitly account for channel non-stationarity and hardware impairments—will be essential for bridging the gap between theoretical promise and engineering reality.

Table 3 summarizes the end-to-end communication approaches surveyed in this section.

## 3. Core Challenges and Solution Pathways for AI-Enabled Physical Layer

Despite the significant performance gains and potential paradigm innovations brought by deep learning to the physical layer of wireless communications, several profound challenges arise from its data-driven nature and the rigid requirements of communication systems. This section delves into five major core challenges currently faced in the field—interpretability, data dependence, computational complexity, privacy and security in distributed learning, and system-level performance-overhead trade-offs—while also systematically reviewing the feasible technical solutions proposed by the research community. These challenges cut across all three stages of AI integration surveyed in this paper, though their specific manifestations and urgency vary across module-level replacement, system-level reconstruction, and network-level native intelligence.

A comprehensive survey by Mengistu et al. [31] traced the evolution of learning-based PHY layer approaches from conventional ML to foundation models, aligning with the three-stage taxonomy proposed in this paper.

### 3.1. Interpretability Dilemma

Deep learning models, especially complex deep networks, are often considered “black-boxes” due to their lack of transparency in decision-making processes [14]. This directly conflicts with the engineering principles of high reliability, debuggability, and certifiability required in communication systems. When AI-based receivers make errors, engineers cannot trace the fault’s root cause through mathematical derivation, as with traditional algorithms, creating substantial obstacles for system maintenance and standardization.

**Figure 6:**
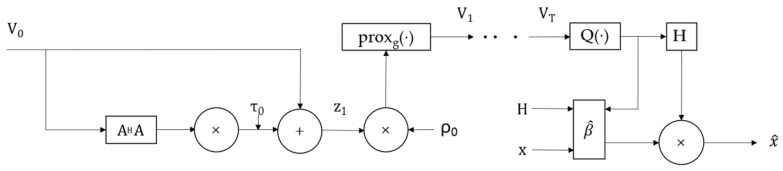
Computational graph of the deep unfolding MU-MIMO 1-bit precoding algorithm. Adapted from [33].

Introducing explainable AI (XAI) techniques, such as visualization methods to highlight the input signal features relied upon by neural networks during decision-making, provides an intuitive tool for understanding model behavior [32]. However, a more fundamental solution is to develop architectures that integrate both model-driven and data-driven approaches. The research by Balatsoukas-Stimming and Studer [33] identifies deep unfolding networks as a representative solution to this challenge. As shown in Figure 6, in an unfolded MU-MIMO precoder, the iterative algorithm’s workflow is converted into a transparent neural network layer, where only a few key parameters need to be learned from data. This allows each layer of the network to have clear physical meaning, significantly enhancing the model’s transparency and interpretability. By optimizing these parameters through end-to-end training, the network typically achieves faster convergence and better performance than using heuristic parameter settings [18].

In addition to embedding prior knowledge into the model structure to improve interpretability, another approach is to build reliability and security assurance mechanisms for AI models at the system design and evaluation levels. This is particularly critical for deploying AI-driven communication subsystems in safety-critical applications such as healthcare and industrial control. For example, in AI-native network slicing for 6G healthcare IoT systems, researchers emphasize that interpretability (Explainability) must be addressed as a core challenge. Due to the “black-box” nature of deep reinforcement learning (RL) and large AI models (LAM), their decision logic lacks transparency, potentially undermining trust among clinicians and regulatory bodies. Future research must explore integrating XAI technologies into digital twins (DT) and LAMs to provide decision-making rationale and meet stringent requirements for transparency and accountability in safety-critical environments [34]. This shows that interpretability is not merely an algorithmic concern but a practical engineering challenge involving system design, human–machine interaction, and standardization.

Complementing deep unfolding approaches, recent work on large AI models (LAMs) has demonstrated robust generalization and multitask capabilities for wireless physical layer tasks, including channel estimation, signal detection, and beamforming [35].

The interpretability deficit manifests differently across the core physical-layer tasks surveyed in Section 2. In channel estimation, the opacity of CNN and RNN architectures obscures the relationship between network topology and estimation accuracy, hindering principled architecture design. In signal detection, although deep unfolding networks offer improved transparency by embedding algorithmic structure, the physical significance of learned parameters—such as step sizes and shrinkage thresholds—remains opaque, precluding the theoretical performance guarantees that accompany classical detectors. The problem is most pronounced in end-to-end communication systems, where learned waveforms fall entirely outside the analytical framework of conventional communication theory. Consequently, the development of task-specific explainability methods is imperative for advancing AI-driven physical-layer technologies toward operational deployment.

### 3.2. Data Dependence and Generalization Capability

The performance of deep learning models is highly dependent on the consistency between the training data and the statistical distribution of the target application environment [36,37]. However, wireless communication environments are highly dynamic and heterogeneous, and obtaining a comprehensive annotated dataset that covers all possible channel conditions, hardware impairments, and interference patterns is both costly and impractical. As a result, models trained in one specific scenario may suffer a catastrophic performance drop when exposed to different environments, exhibiting poor generalization ability [2].

The generalization capability of deep learning at the wireless physical layer faces triple challenges from time-varying complex channels, multi-user interference, and hardware constraints, and this issue is particularly prominent in multi-antenna systems [37]. Siddiqui et al. [38] point out that multi-antenna technologies, as a core enabler of B5G/6G systems, commonly suffer from generalization deficiencies in AI-driven signal processing solutions. Models trained under specific channel scenarios may exhibit significant performance degradation under dynamic channel variations, fluctuating user numbers, or limited hardware resources.

**Figure 7:**
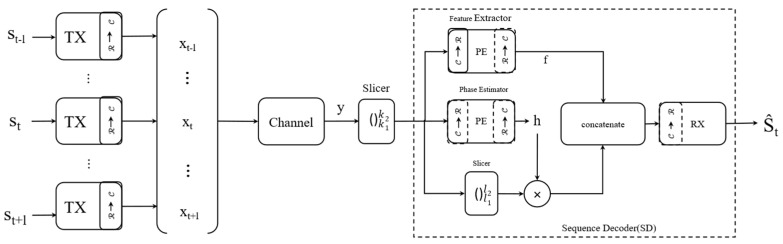
Random channel model for end-to-end training. Adapted from [26].

First, transfer learning has emerged as an effective strategy. Morocho-Cayamcela et al. [39] pointed out that the key idea behind transfer learning is to pre-train a model on a source domain (such as a precise random channel model) and then fine-tune it using a small number of new samples from the target domain, facilitating rapid knowledge transfer and adaptation (Figure 7).

A carefully designed random channel model is the foundation for successful transfer learning. By exposing the neural network to a variety of key impairments (e.g., timing offsets, CFO) in simulation training, the model learns signal representations that are inherently robust to these perturbations. Essentially, this serves as a systematic form of data augmentation that broadens the distribution of the training data, thereby providing a solid foundation for stable performance in real-world environments. This approach shows that the “high-fidelity simulation + transfer learning” framework can substantially alleviate the need for massive real annotated datasets, offering a viable path to improve a model’s generalization ability across different environments.

Second, advanced paradigms such as meta-learning and self-supervised learning have also shown immense potential. Meta-learning focuses on enabling models to “learn how to learn,” optimizing their initial parameters or learning rules to quickly adapt to new tasks with a minimal amount of new samples [40]. Research by Ericsson et al. [41] demonstrated that self-supervised learning, through the design of proxy tasks, allows deep networks to learn robust and generalized feature representations from vast amounts of unlabeled channel data, significantly reducing the dependence on costly labeled data.

Third, three categories of robustness optimization pathways have been proposed for multi-antenna systems. Besides transfer learning, distributed learning and federated learning frameworks are adopted to enable collaborative training across multiple nodes, thereby avoiding overfitting to data from a single scenario and adapting to the distributed architecture of cell-free mMIMO systems. Additionally, lightweight AI models are designed to reduce hardware complexity while minimizing model dependence on specific hardware environments [38].

**Figure 8:**
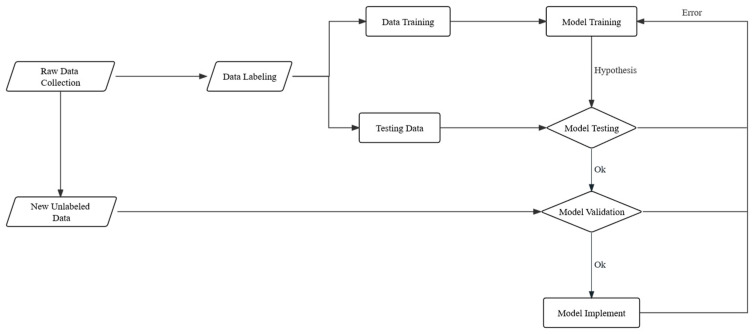
Automatic adaptation process of machine learning algorithms. Adapted from [39].

Improving environmental adaptability requires innovation in both learning paradigms and data foundations. As illustrated in Figure 8, even traditional supervised learning models can gradually improve adaptability to dynamic and heterogeneous wireless environments through a closed-loop process of “data collection—error feedback—parameter update.” Advanced paradigms such as meta-learning and transfer learning further compress the sample size and iteration time required for adapting to new environments, essentially representing an efficiency optimization of the adaptive logic shown in Figure 8. On the other hand, constructing large-scale, high-fidelity channel datasets constitutes the cornerstone for strengthening the training foundation and enhancing generalization performance [5]. High-quality datasets can reduce the discrepancy between initial model training and real-world environments, thereby mitigating the impact of distribution shift on generalization performance at its source.

The generalization challenge affects different physical-layer tasks with varying severity. Channel estimation methods are primarily sensitive to discrepancies between training and deployment channel models. Signal detection algorithms must contend with distributional shifts arising from fluctuations in user populations, modulation schemes, and antenna geometries. End-to-end autoencoder systems face the most acute generalization challenge, as they jointly optimize the entire communication link and are therefore vulnerable to any distributional shift along the signal path. This differential vulnerability explains why AI-based channel estimators and detectors have progressed further toward practical implementation, whereas end-to-end systems remain predominantly confined to simulation studies.

### 3.3. Computational Complexity and Real-Time Constraints

The complexity of deep learning models, particularly those used for large-scale MIMO detection or high-dimensional channel estimation, results in massive computational and memory access overheads. In physical-layer signal processing, especially for ultra-reliable low-latency communication (URLLC) applications, there are strict latency and energy constraints [37]. Deploying large, unoptimized models on resource-constrained devices or edge hardware is impractical, as it may not meet the real-time processing requirements.

Model compression and lightweight techniques have been identified as key solutions to address this issue. Techniques such as network pruning, weight quantization, and knowledge distillation can significantly reduce the size of models and the number of computations required while maintaining acceptable performance losses [42]. Moreover, research by Elsken et al. [43] highlights the potential of neural architecture search (NAS) techniques, which automate the design of specialized network architectures that achieve the best balance between performance and computational complexity.

At the hardware level, Reuther et al. [44] confirmed that specialized AI accelerators and optimized inference frameworks are essential for meeting the real-time demands of physical-layer processing. These technologies provide the underlying computational support for efficiently deploying deep learning models in resource-constrained devices.

The ultimate goal of these lightweight and acceleration techniques is to meet the stringent millisecond or even sub-millisecond processing delay requirements for physical-layer tasks. For example, in the automatic modulation classification (AMC) task, the CAIC-Net model, designed for low complexity, has been carefully evaluated for inference efficiency [45]. Experimental results showed that a well-designed lightweight network can achieve microsecond-level inference time while maintaining high accuracy, proving that AI models can indeed be integrated into real-time signal processing chains to meet the computational and latency constraints of physical-layer tasks.

Additionally, Hu et al. [46] proposed the IMS 2.0 framework as a practical example of low-complexity control. The framework’s control strategy module uses simplified computational paths and adaptive algorithms to reduce computational overhead, ensuring that real-time requirements are met without the need for dedicated hardware acceleration. This dynamic optimization module balances the load during inference, ensuring that latency remains within the millisecond range, even in high-interference and dynamic channel environments. This collaboration between model lightweighting and hardware acceleration provides a crucial reference for the deployment of AI-driven systems in resource-constrained edge devices.

While large AI models (LAMs) have demonstrated promising generalization and multitask capabilities for physical-layer tasks [35], their substantial computational and memory footprints pose significant challenges for practical deployment. The inference of billion-parameter models demands GPU clusters or specialized accelerators that are incompatible with the resource budgets of edge devices and base stations. Even with model compression techniques such as pruning and quantization, the computational overhead of LAMs remains orders of magnitude higher than that of task-specific lightweight networks. Consequently, the application of LAMs in latency-sensitive physical-layer scenarios requires careful consideration of the accuracy–efficiency trade-off, and their current role is more appropriately positioned as a research exploration rather than a near-term deployable solution.

### 3.4. Privacy and Security Challenges in Distributed Learning

Distributed learning architectures, such as federated learning, provide an effective approach for protecting user data privacy by avoiding the uploading of raw data. However, the collaborative training process itself introduces new security vulnerabilities [47,48]. During the exchange of model parameters or gradients, the system may be exposed to threats such as model inversion attacks and data poisoning attacks. Malicious participants may infer sensitive user information from shared model updates or upload carefully crafted malicious data to compromise the integrity and reliability of the global model.

To address this challenge, the deep integration of cryptographic tools and machine learning algorithms has become an inevitable trend [48]. Differential privacy techniques add mathematically calibrated random noise to model updates, thereby effectively preventing reverse inference of individual data under a quantifiable privacy budget. Meanwhile, robust aggregation algorithms are designed to identify and exclude abnormal local model updates, becoming necessary measures to defend against poisoning attacks and ensure the security of collaborative learning.

In emerging 6G-enabled application scenarios, the trade-off between data privacy and model security becomes more complex. In intelligent healthcare systems based on digital twins (DT), patient DTs contain highly sensitive physiological data and health status information, and their synchronization and training processes impose extremely stringent privacy requirements. Applying federated learning alone may be insufficient to counter threats such as model inversion attacks. Therefore, related studies indicate that hybrid privacy-preserving frameworks combining federated learning, homomorphic encryption, and differential privacy should be explored. In addition, blockchain technology may be introduced into sensitive data flows to enable auditable accountability mechanisms [34]. This reveals a fundamental tension within the field: the inherent trade-off between the depth of intelligence and the strength of privacy protection. Future privacy-preserving solutions must result from refined trade-offs among model performance, learning efficiency, privacy strength, and security overhead under specific application scenarios, rather than serving as universal solutions.

### 3.5. System-Level Evaluation and Performance–Overhead Trade-Off

To place these trade-offs on a more concrete footing, consider the following representative data points drawn from the literature surveyed in this paper. While these figures are reported under different experimental conditions and are not directly comparable in a rigorous benchmarking sense, they provide useful order-of-magnitude illustrations of the performance–complexity spectrum. In channel estimation, many existing studies focus on demonstrating the potential of AI models in improving specific communication performance metrics, while generally neglecting the additional system overhead introduced by AI integration [30,49]. Such overhead includes computational power and energy consumption required for model training and inference, air-interface signaling resources for model updates or federated learning, and memory occupation for model storage. Without a comprehensive trade-off analysis between performance gains and resource costs, proposed designs may lack feasibility and cost-effectiveness in practical systems.

Deep learning-enabled semantic communication architectures clearly reveal the intrinsic relationship between performance gains and resource overhead [49]. By employing a semantic encoder to extract task-relevant features and filter redundant data, the architecture significantly improves spectral efficiency and transmission reliability, which constitute the primary performance advantages emphasized in current research. However, the semantic encoder relies on complex deep learning models such as Transformers and CNNs, whose inference processes consume substantial computational resources on edge devices. The training phase further requires massive labeled datasets, and the end-to-end optimization of the JSCC module necessitates storing a large number of model parameters. These computational and storage costs are often overlooked in existing studies. Furthermore, when the architecture is applied to multi-user or distributed scenarios, the air-interface transmission of semantic features increases signaling overhead and latency, further highlighting the inherent trade-off between performance enhancement and system cost.

**Figure 9:**
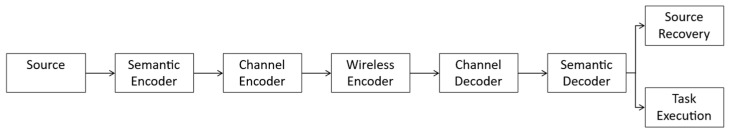
Deep learning-enabled semantic communication system architecture. Adapted from [49].

As shown in Figure 9, the architecture clearly indicates that performance improvements achieved through AI-enabled communication are not without cost. The embedded AI modules introduce multi-dimensional system overhead, including computational complexity, storage consumption, and latency. This perspective reminds researchers that future studies should not solely focus on optimizing individual communication metrics. Instead, a comprehensive “performance–overhead” evaluation framework should be established. While leveraging AI to enhance spectral efficiency and reduce bit error rates, system overhead must be reduced through techniques such as model compression (pruning and quantization) and lightweight architecture design, thereby ensuring practical feasibility.

To place these trade-offs on a more concrete footing, consider the following representative data points drawn from the literature surveyed in this paper. In channel estimation, CNN-based methods such as ComNet [13] complete inference within microseconds, whereas diffusion models [19] require tens to hundreds of iterative denoising steps, translating to inference times on the order of seconds. In signal detection, DetNet [22] compresses what would require tens of classical iterations into approximately ten learned layers, achieving an estimated 3–5× reduction in computational complexity relative to conventional iterative detectors. On the hardware side, FPGA-based implementations of RIS configuration networks have demonstrated a 20× speedup over FP32 GPU implementations [50], illustrating the potential of specialized hardware acceleration. These contrasts demonstrate that lightweight design must be treated as a quantifiable engineering requirement rather than a qualitative goal. Future research should routinely report computational metrics alongside traditional communication performance metrics.

## 4. AI-Enabled New Paradigms and Scenarios for 6G

The vision for sixth-generation (6G) wireless communication systems has evolved beyond simple enhancements in connectivity to the creation of a deeply integrated network that blends the physical and digital worlds. AI will transition from an external optimization tool to the core driving force that redefines network architecture and new service models [4]. These new paradigms mark the entry into Stage III—network-level native intelligence—wherein AI becomes an intrinsic architectural element rather than an externally applied optimization tool.

### 4.1. From AI-Enhanced to AI-Native: A Paradigm Shift

A defining characteristic of 6G that distinguishes it from all preceding generations is that artificial intelligence and machine learning will not merely be applied to optimize existing network functions after the fact; rather, they will be integrated into the system architecture from the ground up as a foundational design principle. In 5G, AI was largely employed as an add-on optimization tool for specific tasks such as beam management, resource scheduling, or channel estimation—enhancing performance within an otherwise conventional network architecture. In contrast, 6G envisions AI as a native capability embedded across all layers of the protocol stack, from the physical layer to the application layer, enabling networks that can perceive, reason, learn, and adapt autonomously. This transition from “AI-enhanced” to “AI-native” represents not an incremental improvement but a fundamental paradigm change in how communication systems are conceived, designed, and operated.

This paradigm shift manifests across multiple dimensions. At the architectural level, it gives rise to AI-native network designs where every network node possesses perception and decision-making capabilities (Section 4.2). At the functional level, it enables the deep convergence of communication, sensing, and computing—three capabilities that have historically been designed and optimized in isolation (Section 4.3). At the physical level, it transforms the wireless propagation environment itself into a programmable resource through intelligent surfaces (Section 4.4). This three-dimensional exposition—spanning architecture, function, and physical environment—is not merely a catalog of 6G technologies; it is organized around the central thesis that AI is transitioning from an external enabler to a foundational design principle, a perspective that distinguishes this survey from prior reviews that treat these topics as separate, standalone subjects. The remainder of this section surveys these three paradigms as distinct yet interrelated expressions of the AI-native vision for 6G.

### 4.2. AI-Native Networks: From Enhanced to Embedded Intelligence

This paradigm exemplifies the architectural dimension of the AI-native transition. In traditional networks, AI functions as an “add-on” module that solves specific problems—this is precisely the paradigm that 6G aims to transcend. Under the AI-native design philosophy, machine learning capabilities are deeply embedded into the atomic operations and resource scheduling cores of the protocol stack, endowing the network with the ability to perceive, decide, and evolve as a living entity [8,51].

This fundamental shift will give rise to new architectural designs. Future wireless access networks may evolve into a distributed agent collaboration system, where each network node (such as a base station, intelligent reflecting surface, or terminal) is viewed as an intelligent agent with local perception and decision-making capabilities. These agents will work together through efficient communication and collaborative learning to achieve global resource optimization, interference management, and mobility control. For instance, Liu et al. [47] pointed out that federated learning and other distributed machine learning paradigms provide a feasible framework for collaborative model training and decision-making across network nodes, serving as a key enabler for realizing distributed intelligence. This architecture transforms the network from a passive service provider that responds to demands into an intelligent platform capable of proactively predicting business needs and dynamically allocating resources—connecting, computing, and providing intelligence in an integrated manner.

**Figure 10:**
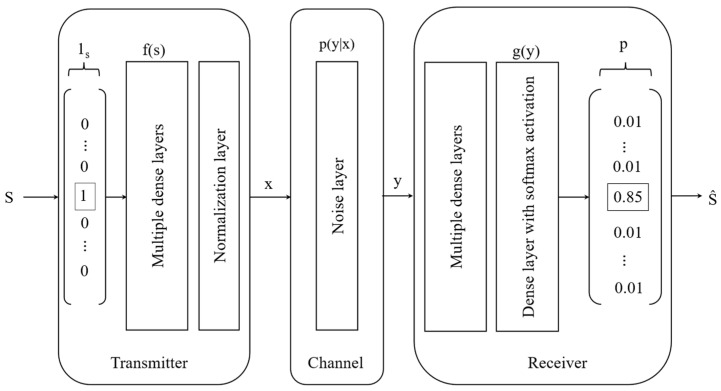
Autoencoder-based end-to-end communication system architecture. Adapted from [1].

Figure 10 shows an early example of this “native intelligence” architecture using an autoencoder framework proposed by Alnaseri et al. [7]. It redefines the communication system as a single, optimizable neural model, a concept that aligns with the vision of AI-native 6G networks.

### 4.3. Integrated Sensing, Communication, and Intelligent Computing: Deep Coordination of Multi-Dimensional Resources

At the functional level, the AI-native paradigm enables the deep integration of communication, sensing, and computing functionalities—three capabilities that have historically been designed and optimized in isolation. A revolutionary paradigm of 6G lies in their unification through joint resource orchestration [52]. The objective is not a simple functional stacking, but rather a fundamental joint design across signal, spectrum, hardware, and protocol layers, so as to support application scenarios such as smart cities and autonomous driving that impose extremely high comprehensive demands on information acquisition, transmission, and processing.

The technical core of integrated sensing, communication, and computing (ISAC) resides in the dual fusion of signals and resources. At the signal level, the same wireless waveforms and hardware infrastructure are employed to simultaneously accomplish data transmission and environmental sensing, while advanced signal processing algorithms are used to separate communication and sensing information streams. At the resource level, computational resources, storage resources, and communication resources (e.g., spectrum, time slots, and beams) are abstracted and managed in a unified manner.

Mao et al. [9] point out that the essence of rate-splitting multiple access (RSMA) lies in enabling intelligent and dynamic partitioning of multi-user resources in both power and spatial domains at the physical layer through advanced signal processing and interference management. This provides fundamental technical insights for multidimensional resource scheduling in ISAC scenarios. AI algorithms, particularly deep reinforcement learning, have become key enablers for cross-domain dynamic scheduling, allowing optimal trade-offs among communication rate, sensing accuracy, and computational load based on real-time task requirements.

For example, in dense multi-user MIMO scenarios toward 6G, multi-agent reinforcement learning frameworks have been adopted, where each base station acts as an intelligent agent. Through collaborative learning, agents dynamically optimize 3D beamforming weights and user association strategies. This approach jointly improves spectral efficiency and energy efficiency while effectively managing inter-user interference, demonstrating the powerful capability of AI in complex resource coordination problems [49,53,54].

One of the core challenges in ISAC lies in the bidirectional adaptation of modulation design—communication aims to minimize the bit error rate (BER), while sensing requires optimized waveform autocorrelation properties. Traditional solutions struggle to achieve a dynamic balance between these objectives. To address this contradiction, a deep neural network-based end-to-end autoencoder framework has been proposed to optimize constellation mapping and demapping processes in ISAC systems [53].

The framework consists of encoding and decoding modules at the transmitter and receiver, and employs a composite loss function for joint training. It dynamically adjusts the geometric distribution and transmission probabilities of constellation points. Leveraging OFDM waveforms and pulse transmission mechanisms, under a 16-point constellation configuration, the proposed scheme achieves significant improvements in peak sidelobe level ratio compared with conventional modulation methods, while maintaining BER performance comparable to mainstream modulation schemes. In several traditional modulation scenarios, it further achieves signal-to-noise ratio gains. Meanwhile, the framework features remarkably low complexity: both transmitter and receiver networks consist of two fully connected layers, and the inference efficiency and latency fully satisfy the real-time deployment requirements of ISAC systems [53].

For millimeter-wave multi-user MIMO scenarios, a deep reinforcement learning-based angular-domain hybrid precoding framework has been proposed to achieve joint optimization of energy efficiency and spectral efficiency through reward-maximizing beam training [54]. The framework dynamically adjusts the number of active RF chains using angular-domain information, thereby reducing beam training overhead and channel estimation complexity. The deep reinforcement learning agent learns optimal precoding strategies through interaction with the environment, with a reward function that jointly considers performance metrics and training overhead to maximize long-term gains. Simulation results demonstrate significant spectral efficiency improvement compared with conventional methods. Even with increased total power consumption, the framework achieves a favorable balance between performance and efficiency, providing a concrete implementation pathway for multidimensional resource orchestration in ISAC scenarios [54].

Beyond these deep learning and reinforcement learning approaches, generative artificial intelligence (GAI) has recently emerged as a promising paradigm for ISAC. Leblebici and Çalhan [55] reviewed the role of GAI in overcoming data scarcity and optimizing resource allocation in 6G ISAC systems, highlighting its potential to address challenges across physical, data link, and network layers. A comprehensive survey by Zhang et al. [56] provided an overview of intelligent ISAC, covering its motivation, typical applications, recent trends, and challenges, complementing the GAI-ISAC discussion in this section.

### 4.4. Reconfigurable Intelligent Surfaces and Active Wireless Environment Shaping

At the physical level, the AI-native paradigm transforms the wireless propagation environment itself from a passive medium into a programmable resource. To address challenges such as severe coverage attenuation and high energy consumption in high-frequency bands, 6G introduces reconfigurable intelligent surfaces (RIS) as a novel foundational technology. The integration of RIS with AI marks a paradigm shift from “adapting to the channel” to “shaping the channel.”

RIS consists of a large number of programmable electromagnetic meta-material elements capable of intelligently adjusting the phase and amplitude of incident wireless signals through AI-driven algorithms, thereby actively constructing favorable wireless propagation environments. However, the introduction of RIS also brings unprecedented high-dimensional joint optimization challenges. The phase configuration of RIS must be jointly optimized with base station transmit beamforming and user-side reception strategies.

To address the core challenge of channel estimation in RIS-assisted systems, systematic deep learning-based solutions have been developed. For different RIS-assisted scenarios, including massive MIMO, millimeter-wave, and multi-user systems, researchers have designed various data-driven and model-driven approaches—such as deep compressive sensing, attention networks, and image super-resolution networks—to cope with the high dimensionality and overhead introduced by the passive characteristics of RIS [12].

Solving such complex, high-dimensional, and non-convex optimization problems highlights the fundamental advantage of hybrid model-driven and data-driven methods [24]. For example, deep unfolding architectures can map iterative optimization steps of RIS configuration algorithms into neural network layers and use data to optimize internal parameters. This enables real-time computation of optimal joint control strategies with reduced computational complexity and improved efficiency, making the wireless environment itself a programmable intelligent resource.

Martín-Martín et al. [50] proposed a customized convolutional neural network (CNN) tailored for edge computing to solve the optimal phase configuration problem of RIS. The network takes the radiation pattern corresponding to a target steering angle as input and outputs a 15 × 15 1-bit RIS configuration by learning the mapping relationship. After training, the classification accuracy reaches 98.88%.

To verify deployment feasibility, the authors compared implementation performance across four types of edge devices: CPU, GPU, TPU, and FPGA. Results show that the FPGA-based solution achieves the best performance—20 times faster than the FP32 GPU implementation and nearly 3 times faster than the INT8-quantized TPU implementation. Moreover, with the aid of high-level synthesis (HLS) tools, efficient deployment can be achieved without designing dedicated accelerators. This synergistic design of “AI algorithms + specialized hardware” provides hardware support for real-time scheduling of RIS in edge scenarios, enabling practical engineering realization of active wireless environment shaping.

## 5. Conclusions

This paper has provided a systematic review of research progress in deep learning-empowered wireless physical-layer communications, highlighting the paradigm evolution from model-driven approaches to data-driven methodologies and further toward model–data fusion [10,24].

### 5.1. Summary of Main Findings

Viewed through the lens of the three-stage evolutionary framework articulated in this paper, a clear developmental trajectory emerges. Stage I (module-level replacement) has yielded tangible performance gains in channel estimation and signal detection, with several techniques now approaching practical deployment. Stage II (system-level reconstruction), embodied by end-to-end autoencoder architectures, offers the theoretical possibility of transcending conventional performance limits but remains constrained by challenges in interpretability, generalization, and hardware compatibility. Stage III (network-level native intelligence) delineates the aspirational vision for 6G, wherein AI becomes an integral component of network architecture rather than an externally applied tool. Understanding this evolutionary logic provides a structured view of current research and clarifies the key technical transitions ahead.

Building upon this foundation, we critically investigated five fundamental challenges currently constraining the field—interpretability, data dependence, computational complexity, privacy and security in distributed learning, and system-level performance-overhead trade-offs—and comprehensively reviewed state-of-the-art solution pathways such as deep unfolding, transfer learning, meta-learning, model lightweighting, federated learning, differential privacy, and co-design evaluation frameworks. In particular, recent advances in generative AI and diffusion models [10,19,20,55] have shown great potential in channel estimation and ISAC, although their practical deployment remains constrained by substantial inference costs that render them unsuitable for latency-critical applications without further optimization, offering new ways to overcome data limitations and dynamic environment challenges.

It must be recognized that, despite the impressive performance gains enabled by deep learning at the wireless physical layer, several fundamental limitations persist. First, the generalization capability of most AI-driven physical-layer methods remains fragile—models trained under specific channel statistics frequently exhibit performance collapse when deployed in even slightly altered environments, a vulnerability that is particularly severe for end-to-end autoencoder architectures. Second, the trade-off between performance and computational complexity has yet to be systematically resolved; high-performance approaches such as diffusion models incur inference costs that are prohibitive for latency-sensitive applications, while lightweight alternatives sacrifice accuracy under challenging channel conditions. Third, the interpretability gap between data-driven and model-based methodologies impedes standardization, complicates system-level verification, and undermines trust in safety-critical deployments. Fourth, the predominant reliance on simulation-based validation, often neglecting hardware impairments and RF non-idealities, leaves the practical feasibility of many proposed techniques an open question.

### 5.2. Future Perspectives and Open Challenges

The analytical framework proposed in this work centers on the technical logic and evolutionary pathway of physical-layer intelligence, taking the model–data relationship as the conceptual thread that runs throughout the study. We argue that hybrid intelligence—organically integrating communication-theoretic priors with the expressive capacity of neural networks—constitutes a fundamental architectural pathway rather than a compromise between purely model-driven and purely data-driven paradigms. Furthermore, we establish the comprehensive performance–overhead trade-off as a core criterion for system-level evaluation of AI-enabled communication models, demonstrating that emerging paradigms such as semantic communication inevitably face fundamental trade-offs between spectral efficiency gains and additional computational, storage, and signaling costs. In parallel, we incorporate the inherent trade-offs among privacy, performance, and security in distributed intelligence into the analytical framework, emphasizing both the necessity and the complexity of integrating differential privacy, federated learning, and homomorphic encryption mechanisms.

Looking ahead, intelligent wireless physical-layer research for 6G stands at a pivotal turning point, transitioning from AI-enabled communication toward genuine AI–communication symbiosis. Several open challenges define this transition. The first is the coordination mechanism between models and data: how to systematically combine communication-theoretic priors with data-driven learning to achieve both interpretability and adaptability remains an unresolved question. The second concerns trustworthy architectures for distributed intelligence: as networks become more autonomous, ensuring robustness, security, and accountability across decentralized AI components becomes essential. The third involves the practical realization of semantic communication: moving from proof-of-concept demonstrations to standardized, interoperable systems requires breakthroughs in both theory and engineering. The fourth is the establishment of comprehensive system-level evaluation frameworks that go beyond traditional communication metrics to incorporate computational cost, energy efficiency, and deployment feasibility.

Addressing these challenges requires close collaboration across communication theory, artificial intelligence, electromagnetic metamaterials, and chip architecture design. When physical-layer algorithm designers and hardware architects collaboratively optimize inference latency, when channel modeling experts and metamaterial researchers jointly program the wireless propagation environment, and when semantic information theory meaningfully converges with deep learning at the coding-theoretic level, the vision of natively intelligent 6G networks can be firmly grounded at the physical-layer foundation. We hope this survey provides a useful reference for researchers working toward the practical deployment of AI in next-generation physical-layer systems.

## Figures and Tables

**Table 1:** Summary of representative deep learning methods for channel estimation.

Method	Core Principle	Reported Advantage	Primary Limitation	Complexity	Ref.
ComNet (CNN)	Time-frequency grid as image; CNN denoising	Low complexity, robust local feature extraction	Limited long-range dependency modeling	Low	[13]
ChanEstNet (RNN/LSTM)	Temporal CSI sequence prediction	Superior tracking in high-mobility scenarios	Gradient vanishing, limited parallelizability	Medium	[15]
RL-MFF-Net (Residual + Fusion)	Super-resolution with multi-path feature fusion	~4 dB gain, low pilot overhead	Complex architecture	Medium	[16]
OAMP-Net2 (Deep Unfolding)	Unrolled iterative algorithm with learnable parameters	Interpretable, fast convergence, parameter-efficient	Algorithm-specific structure	Medium	[18]
DDPM-CE (Diffusion)	Iterative denoising via learned score function	Robust at extremely low SNR	High inference latency, unsuitable for URLLC	High	[19]

**Table 2:** Summary of representative deep learning methods for signal detection.

Method	Core Principle	Reported Advantage	Primary Limitation	Complexity	Ref.
DetNet (Deep Unfolding)	Unrolled projected gradient descent	Near-ML performance, manageable complexity	Sensitive to ill-conditioned channels	Medium	[22]
OAMP-Net (Deep Unfolding)	Unrolled approximate message passing	Theoretical convergence guarantees	Assumes i.i.d. channel conditions	Medium	[18]
OHA/DSE (Classification)	Detection as multi-class classification	Lightweight, edge-device compatible	Class explosion with high-order modulation	Low	[25]

**Table 3:** Summary of representative end-to-end communication methods.

Method	Core Principle	Reported Advantage	Primary Limitation	Complexity	Ref.
Autoencoder (Dörner)	Joint transceiver optimization	Theoretically surpasses modular limits	Poor interpretability and generalization	High	[26]
Deep Unfolding E2E	Modular priors embedded in trainable layers	Balanced interpretability and performance	Complex architecture design	Medium	[24]
Joint Coding-Estimation (Christopoulou)	Unified coding-estimation-classification	Reduced BER in interference-limited bands	Task-specific design	Medium	[30]
E2E-Semantic (Islam)	E2E PHY for multimodal semantic applications	Semantic-aware 6G integration	Niche application domain	Medium	[28]

## Data Availability

No new data were created or analyzed in this study.

## References

[1] O’Shea T., Hoydis J. (2017). An introduction to deep learning for the physical layer. IEEE Trans. Cogn. Commun. Netw..

[2] Wang T., Wen C.-K., Wang H., Gao F., Jiang T., Jin S. (2017). Deep learning for wireless physical layer: Opportunities and challenges. China Commun..

[3] Shah A.F.M.S., Qasim A.N., Karabulut M.A., Ilhan H., Islam M.B. (2021). Survey and performance evaluation of multiple access schemes for next-generation wireless communication systems. IEEE Access.

[4] You X., Wang C., Huang J., Gao X., Zhang Z., Wang M., Larsson E.G. (2021). Towards 6G wireless communication networks: Vision, enabling technologies, and new paradigm shifts. Sci. China Inf. Sci..

[5] Wang C.-X., You X., Gao X., Zhu X., Li Z., Zhang C., Hanzo L. (2023). On the road to 6G: Visions, requirements, key technologies and testbeds. IEEE Commun. Surv. Tutor..

[6] Chataut R., Akl R. (2020). Massive MIMO systems for 5G and beyond networks—Overview, recent trends, challenges, and future research direction. Sensors.

[7] Alnaseri O., Alzubaidi L., Himeur Y., Ala’anzy M.A., Timmermann J., Gismalla M.S.M. (2025). A Review on Deep Learning Autoencoder in the Design of Next-Generation Communication Systems. arXiv.

[8] Jiang C., Zhang H., Ren Y., Han Z., Chen K.-C., Hanzo L. (2017). Machine learning paradigms for next-generation wireless networks. IEEE Wirel. Commun..

[9] Mao Y., Dizdar O., Clerckx B., Schober R., Popovski P., Poor H.V. (2022). Rate-splitting multiple access: Fundamentals, survey, and future research trends. IEEE Commun. Surv. Tutor..

[10] Van Huynh N., Wang J., Du H., Hoang D.T., Niyato D., Nguyen D.N., Kim D.I., Letaief K.B. (2024). Generative AI for Physical Layer Communications: A Survey. IEEE Trans. Cogn. Commun. Netw..

[11] Wen C.-K., Shih W.-T., Jin S. (2018). Deep learning for massive MIMO CSI feedback. IEEE Wirel. Commun. Lett..

[12] Lv C., Luo Z. (2023). Deep Learning for Channel Estimation in Physical Layer Wireless Communications: Fundamental, Methods, and Challenges. Electronics.

[13] Gao X., Jin S., Wen C.-K., Li G.Y. (2018). ComNet: Combination of Deep Learning and Expert Knowledge in OFDM Receivers. IEEE Commun. Lett..

[14] Zhang C., Patras P., Haddadi H. (2019). Deep learning in mobile and wireless networking: A survey. IEEE Commun. Surv. Tutor..

[15] Liao Y., Hua Y., Dai X., Yao H., Yang X. (2019). ChanEstNet: A deep learning-based channel estimation for high-speed scenarios. Proceedings of the ICC 2019—2019 IEEE International Conference on Communications (ICC), Shanghai, China, 20–24 May 2019.

[16] Zheng X., Liu Z., Liang J., Wu Y., Chen Y., Zhang Q. (2022). Residual Learning and Multi-Path Feature Fusion-Based Channel Estimation for Millimeter-Wave Massive MIMO System. Entropy.

[17] Soltani M., Pourahmadi V., Mirzaei A., Sheikhzadeh H. (2019). Deep learning-based channel estimation. IEEE Commun. Lett..

[18] He H., Wen C.-K., Jin S., Li G.Y. (2020). Model-driven deep learning for MIMO detection. IEEE Trans. Signal Process..

[19] Zhou X., Liang L., Zhang J., Jiang P., Li Y., Jin S. (2026). Generative Diffusion Models for High Dimensional Channel Estimation. arXiv.

[20] Fu H., Si W., Liu R. (2025). Conditional Denoising Diffusion-Based Channel Estimation for Fast Time-Varying MIMO-OFDM Systems. Digit. Signal Process..

[21] Ying K., Gao Z., Chen S., Quek T.Q.S., Poor H.V. (2026). Generative Diffusion Model Driven Massive Random Access in Massive MIMO Systems. arXiv.

[22] Wang H., Wen F., Wang X., Du W., Gui G. (2026). Hierarchical Channel Estimation for Near-Field Spatial Non-Stationary Channels: A Pre-Selection and Multi-Level Dynamic Threshold Strategy. IEEE Trans. Cogn. Commun. Netw..

[23] Samuel N., Diskin T., Wiesel A. (2017). Deep MIMO detection. Proceedings of the 2017 IEEE 18th International Workshop on Signal Processing Advances in Wireless Communications (SPAWC), Sapporo, Japan, 3–6 July 2017.

[24] Deka S., Deka K., Thanh Nguyen N., Sharma S., Bhatia V., Rajatheva N. (2026). Comprehensive Review of Deep Unfolding Techniques for Next-Generation Wireless Communication Systems. IEEE Internet Things J..

[25] Ibarra-Hernández R.F., Castillo-Soria F.R., Gutiérrez C.A., Del-Puerto-Flores J.A., Acosta-Elias J., Rodriguez-Abdala V.I., Palacios-Luengas L. (2025). Efficient Deep Learning-Based Detection Scheme for MIMO Communication Systems. Sensors.

[26] Dörner S., Cammerer S., Hoydis J., ten Brink S. (2018). Deep learning based communication over the air. IEEE J. Sel. Top. Signal Process..

[27] Elfiky A., Rezki Z., Cortez J., Boumhaout Y., Xia A., Celik A., Kaddoum G. (2026). End-to-End Deep Learning in Wireless Communication Systems: A Tutorial Review. arXiv.

[28] Islam N., Shin S. (2024). Deep Learning in Physical Layer: Review on Data Driven End-to-End Communication Systems and Their Enabling Semantic Applications. IEEE Open J. Commun. Soc..

[29] Nachmani E., Marciano E., Lugosch L., Gross W.J., Burshtein D., Be’ery Y. (2018). Deep learning methods for improved decoding of linear codes. IEEE J. Sel. Top. Signal Process..

[30] Christopoulou A., Kosmanos D., Xenakis A., Chaikalis C. (2026). AI-Enabled Autoencoder-Based Physical Layer Design for 6G Communication Systems. Electronics.

[31] Mengistu T.M., Faisal K.M., Kim T., Ullah A., Choi W. (2026). Learning-Based Approaches for Wireless PHY Layers from the Perspective of Conventional Machine Learning to Foundation Models: A Comprehensive Survey. Comput. Sci. Rev..

[32] Sadeghi M., Larsson E.G. (2019). Adversarial attacks on deep-learning-based radio signal classification. IEEE Wirel. Commun. Lett..

[33] Balatsoukas-Stimming A., Studer C. (2019). Deep unfolding for communications systems: A survey and some new directions. 2019 IEEE International Workshop on Signal Processing Systems (SiPS).

[34] Hu H., Song Z., Shi W. (2026). Large AI Model-Enhanced Digital Twin-Driven 6G Healthcare IoE. Electronics.

[35] Guo J., Cui Y., Jin S., Zhang J. (2026). Large AI Models for Wireless Physical Layer. arXiv.

[36] O’Shea T.J., Corgan J., Clancy T.C. (2016). Convolutional Radio Modulation Recognition Networks. arXiv.

[37] Chen M., Challita U., Saad W., Yin C., Debbah M. (2019). Artificial neural networks-based machine learning for wireless networks: A tutorial. IEEE Commun. Surv. Tutor..

[38] Siddiqui M.U.A., Qamar F., Kazmi S.H.A., Hassan R., Arfeen A., Nguyen Q.N. (2023). A Study on Multi-Antenna and Pertinent Technologies with AI/ML Approaches for B5G/6G Networks. Electronics.

[39] Morocho-Cayamcela M.E., Lee H., Lim W. (2019). Machine learning for 5G/B5G mobile and wireless communications: Potential, limitations, and future directions. IEEE Access.

[40] Huang H., Guo S., Gui G., Yang Z., Zhang J., Sari H., Adachi F. (2020). Deep learning for physical-layer 5G wireless techniques: Opportunities, challenges and solutions. IEEE Wirel. Commun..

[41] Ericsson L., Gouk H., Loy C.C., Hospedales T.M. (2022). Self-supervised representation learning: Introduction, advances, and challenges. IEEE Signal Process. Mag..

[42] Han S., Mao H., Dally W.J. (2015). Deep compression: Compressing deep neural networks with pruning, trained quantization and Huffman coding. arXiv.

[43] Elsken T., Metzen J.H., Hutter F. (2019). Neural Architecture Search: A Survey. arXiv.

[44] Reuther A., Michaleas P., Jones M., Gadepally V., Samsi S., Kepner J. (2020). Survey of machine learning accelerators. Proceedings of the IEEE High Performance Extreme Computing Conference (HPEC), Waltham, MA, USA, 22–24 September 2020.

[45] Wu T., Zhu Q., Mao R., Hu C., Wei S. (2026). CAIC-Net: Robust Radio Modulation Classification via Unified Dynamic Cross-Attention and Cross-Signal-to-Noise Ratio Contrastive Learning. Sensors.

[46] Hu X., Cui J., Zhang R., Fang Q. (2026). Reconfigurable Wireless Channel Optimization and Low-Complexity Control Methods Driven by Intelligent Metasurfaces 2.0. Telecom.

[47] Liu Y., Yuan X., Xiong Z., Kang J., Wang X., Niyato D. (2020). Federated learning for 6G communications: Challenges, methods, and future directions. China Commun..

[48] Gündüz D., Qin Z., Aguerri I.E., Dhillon H.S., Yang Z., Yener A., Wong K.K. (2023). Beyond transmitting bits: Context, semantics, and task-oriented communications. IEEE J. Sel. Areas Commun..

[49] Al Janaby A., Al-Rizzo H., Qassim Y. (2026). Enhancing Spectral Efficiency of 6G Downlink Beamforming via Cooperative Multi-Agent Deep Reinforcement Learning. Sensors.

[50] Martín-Martín A., Padial-Allué R., Castillo E., Parrilla L., Parellada-Serrano I., Morán A., García A. (2024). Hardware Implementations of a Deep Learning Approach to Optimal Configuration of Reconfigurable Intelligence Surfaces. Sensors.

[51] Chatzieleftheriou C., Liotou E. (2026). A Survey on AI for 6G: Challenges and Opportunities. IEEE Open J. Commun. Soc..

[52] Tok Y.E., Toprak A.G., Karahan S.N., Mercan O.B., Aydin H.M., Altintas M. (2026). Artificial Intelligence for Next-Generation 6G Technologies and Networks. Discov. Netw..

[53] Yu J., Bai J., Huang J., Wang X., Feng J., Xia F., Zheng Z. (2025). End-to-End Constellation Mapping and Demapping for Integrated Sensing and Communications. Electronics.

[54] Salh A., Alhartomi M.A., Hussain G.A., Jing C.J., Shah N.S.M., Alzahrani S., Alsulami R., Alharbi S., Hakimi A., Almehmadi F.S. (2025). Deep Reinforcement Learning-Driven Hybrid Precoding for Efficient Mm-Wave Multi-User MIMO Systems. J. Sens. Actuator Netw..

[55] Leblebici M., Çalhan A. (2025). Generative Artificial Intelligence for Integrated Sensing and Communication in 6G. Comput. Netw..

[56] Zhang J., Lu W., Xing C., Zhao N., Al-Dhahir N., Karagiannidis G.K., Yang X. (2025). Intelligent integrated sensing and communication: A survey. Sci. China Inf. Sci..

